# The Significant Reduction or Complete Eradication of Subcutaneous and Metastatic Lesions in a Pheochromocytoma Mouse Model after Immunotherapy Using Mannan-BAM, TLR Ligands, and Anti-CD40

**DOI:** 10.3390/cancers11050654

**Published:** 2019-05-11

**Authors:** Veronika Caisova, Liping Li, Garima Gupta, Ivana Jochmanova, Abhishek Jha, Ondrej Uher, Thanh-Truc Huynh, Markku Miettinen, Ying Pang, Luma Abunimer, Gang Niu, Xiaoyuan Chen, Hans Kumar Ghayee, David Taïeb, Zhengping Zhuang, Jan Zenka, Karel Pacak

**Affiliations:** 1 Section on Medical Neuroendocrinology, *Eunice Kennedy Shriver* National Institute of Child Health and Human Development, National Institutes of Health, Bethesda, MD 20814, USA; veronika.caisova@nih.gov (V.C.); bat.150@hotmail.com (L.L.); garima.gupta83@gmail.com (G.G.); ivana.jochmanova@gmail.com (I.J.); abhishek.jha@nih.gov (A.J.); ondrej.uher@nih.gov (O.U.); huynht@mail.nih.gov (T.-T.H.); ying.pang@nih.gov (Y.P.); luma.abunimer@nih.gov (L.A.); 2 Department of Medical Biology, Faculty of Science, University of South Bohemia, Ceske Budejovice 37005, Czech Republic; jzenka@gmail.com; 3 Laboratory of Pathology, National Cancer Institute, National Institutes of Health, Bethesda, MD 20814, USA; markku.miettinen@nih.gov; 4 Laboratory of Molecular Imaging and Nanomedicine, National Institute of Biomedical Imaging and Bioengineering, National Institutes of Health, Bethesda, MD 20814, USA; gang.niu@nih.gov (G.N.); shawn.chen@nih.gov (X.C.); 5 Biological Molecular Imaging Section, University of Florida College of Medicine, Gainesville, FL 32603, USA; Hans.Ghayee@medicine.ufl.edu; 6 Department of Nuclear Medicine, La Timone University Hospital, CERIMED, Aix-Marseille University, 13385 Marseille, France; David.TAIEB@ap-hm.fr; 7 Surgical Neurology Branch, National Institute of Neurological Disorders and Stroke, National Institutes of Health, Bethesda, MD 20814, USA; zhengping.zhuang@nih.gov

**Keywords:** pheochromocytoma, paraganglioma, metastatic, immunotherapy, innate immunity, adaptive immunity, toll-like receptor, pathogen-associated molecular patterns, neutrophil, T cell

## Abstract

Therapeutic options for metastatic pheochromocytoma/paraganglioma (PHEO/PGL) are limited. Here, we tested an immunotherapeutic approach based on intratumoral injections of mannan-BAM with toll-like receptor ligands into subcutaneous PHEO in a mouse model. This therapy elicited a strong innate immunity-mediated antitumor response and resulted in a significantly lower PHEO volume compared to the phosphate buffered saline (PBS)-treated group and in a significant improvement in mice survival. The cytotoxic effect of neutrophils, as innate immune cells predominantly infiltrating treated tumors, was verified in vitro. Moreover, the combination of mannan-BAM and toll-like receptor ligands with agonistic anti-CD40 was associated with increased mice survival. Subsequent tumor re-challenge also supported adaptive immunity activation, reflected primarily by long-term tumor-specific memory. These results were further verified in metastatic PHEO, where the intratumoral injections of mannan-BAM, toll-like receptor ligands, and anti-CD40 into subcutaneous tumors resulted in significantly less intense bioluminescence signals of liver metastatic lesions induced by tail vein injection compared to the PBS-treated group. Subsequent experiments focusing on the depletion of T cell subpopulations confirmed the crucial role of CD8^+^ T cells in inhibition of bioluminescence signal intensity of liver metastatic lesions. These data call for a new therapeutic approach in patients with metastatic PHEO/PGL using immunotherapy that initially activates innate immunity followed by an adaptive immune response.

## 1. Introduction

Pheochromocytomas (PHEOs) and paragangliomas (PGLs) are rare catecholamine-producing neuroendocrine/neural crest cell tumors arising from the adrenal medulla and extra-adrenal paraganglia, respectively [1]. Approximately 10–30% of all PHEOs/PGLs become metastatic and therapeutic options for treating metastatic disease are limited [2]. Therefore, efforts to find new and more effective therapies are urgently needed for patients with either inoperable or metastatic PHEO/PGL.

Immunotherapy, in which immune cells of a patient are used to attack and subsequently eliminate tumor cells, is currently one of the most extensively studied therapeutic approaches in cancer research [3,4]. Not much is known about the interaction between PHEO/PGL and a patient’s immune system. Overall, PHEO/PGL can be considered as immunologically “cold” compared to other cancer types because of their lack of leukocyte fraction, low amount of neoantigens, and low somatic sequence mutation rate [5,6,7]. To date, only one experimental study proposed the use of immunotherapy in PHEO/PGL, specifically chromogranin A, as a potential treatment target [8]. In this study, immunization with chromogranin A peptides induced the production of cytotoxic T cells with subsequent elimination of chromogranin A expressing PHEO cells. Moreover, chromogranin A peptides were suggested as a potential anti-tumor vaccine in PHEO patients with risk of metastatic disease [8]. Recently, two clinical trials focusing on the application of checkpoint inhibitors (specifically nivolumab, ipilimumab, and pembrolizumab) in rare tumors, including PHEO/PGL, were initiated and are in the stage of patient recruitment (ClinicalTrials.gov Identifier: NCT02834013 and NCT02721732).

Current immunotherapeutic approaches to cancer treatment are based either on the activation of innate or more frequently adaptive immunity [9,10,11]. The innate immune system is well conserved, and its response is uniform, robust, and short-lasting, but it can well contribute to cancer treatment and the elimination of metastatic lesions [12,13,14]. Recently, immunotherapy based on the activation of innate immunity via pathogen-associated molecular patterns (PAMPs) has been tested in melanoma, which is also a neural crest tumor. Intratumoral application of PAMPs, particularly ligands stimulating phagocytosis and toll-like receptor (TLR) ligands, resulted in the complete elimination of over 80% of subcutaneous tumors in the B16-F10 melanoma mouse model [15,16,17].

**Figure 1:**
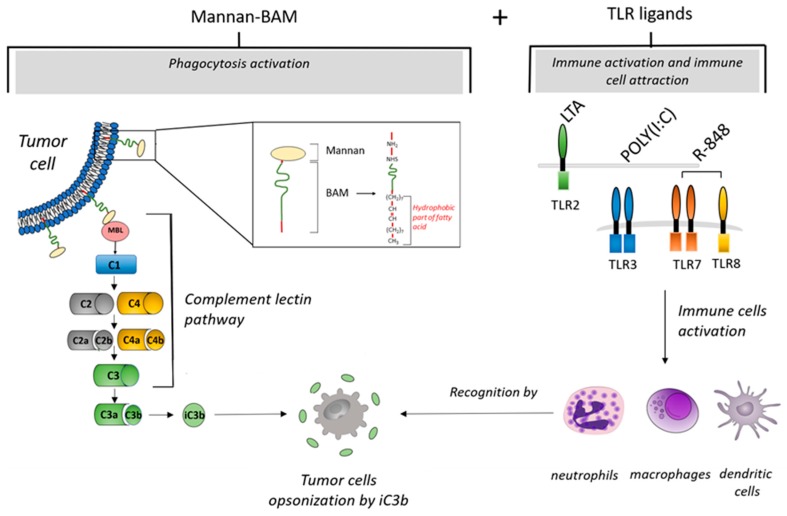
Mechanisms of tumor cell elimination during immunotherapy based on intratumoral application of mannan-BAM+TLR ligands (MBT). After intratumoral application of mannan with a biocompatible anchor for membrane-BAM, the hydrophobic part of BAM is incorporated into the lipid bilayer of tumor cells. Mannan attached to membranes activates innate immunity by the interaction of mannan with mannan binding lectin (MBL). This interaction initiates activation of the complement lectin pathway. This results in iC3b production and opsonization of tumor cells followed by migration of immune cells (macrophages, dendritic cells, or granulocytes) and phagocytosis activation. Further, simultaneous intratumoral application of TLR ligands (resiquimod (R-848), polyinosinic-polycytidylic acid (poly(I:C)), and lipoteichoic acid (LTA)) causes a strong attraction of immune cells (macrophages, dendritic cells, or granulocytes) to the tumor.

Ligands stimulating phagocytosis initiate ingestion of pathogens by phagocytic immune cells [18,19]. PAMP-based immunotherapy uses intratumoral administration of mannan, a simple polysaccharide from *Saccharomyces cerevisiae,* as a ligand stimulating phagocytosis [16]. Mannan recognized by mannan-binding lectin (MBL) activates the complement lectin pathway [20]. This activation results in iC3b molecule production, followed by iC3b tumor cell opsonization [21,22], and consequently their elimination by phagocytes, particularly neutrophils, macrophages, and dendritic cells. In this type of immunotherapy, mannan is bound to a tumor cell membrane by the biocompatible anchor for a membrane called BAM (Figure 1).

TLRs are expressed on the surface of various cells, mainly those belonging to the innate immune system. These receptors recognize their specific ligands and initiate immune system mobilization [23,24,25]. This process is well supported by previous reports showing that the intratumoral application of TLR ligands increased the number of tumor-infiltrating leukocytes in melanoma and renal cell carcinoma mouse models [15,16,17,26]. Resiquimod (R-848), polyinosinic-polycytidylic acid (poly(I:C)), and lipoteichoic acid (LTA) are TLR ligands used in the present study. R-848 is an imidazoquinoline compound with anti-viral effects that activates immune cells via TLR7/TLR8 in humans and TLR7 in mice [27]. Poly(I:C) is a synthetic analog of dsRNA that activates immune cells via TLR3 [28]. LTA is a constituent of the cell wall of Gram-positive bacteria that activates immune cells via TLR2 [29].

Thus, in the present study, we aimed to evaluate the therapeutic effects of intratumorally administered mannan-BAM and TLR ligands (MBT) in a subcutaneous and metastatic mouse PHEO. Specifically, we focused on the initial activation of innate immunity, an assessment of its role in the elimination of PHEO, and the detection of the potential role of subsequent engagement of adaptive immunity in elimination of distant metastatic PHEO organ lesions. This model was established using B6(Cg)-*Tyr^c-2J^*/J mouse strains with subcutaneously and/or intravenously injected experimental PHEO cells called mouse tumor tissue (MTT)-luciferase cells [30,31]. Subsequently, the intratumoral application of MBT resulted in a significantly lower PHEO volume compared to the phosphate buffered saline (PBS)-treated group and in an improvement in mice survival. As neutrophils infiltrated the treated subcutaneous PHEOs, their role in this therapeutic model was established by measuring neutrophil cytotoxic activity and neutrophil-tumor cell interactions with MTT-luciferase and hPheo1 cell lines. An additional boost of MBT with anti-CD40 (this combination is henceforth referred to as MBTA) significantly improved the effect of the MBT application on the survival of experimental mice. Since re-challenge experiment suggested potential engagement of adaptive immunity, the therapy was tested in metastatic PHEO with a positive effect on the bioluminescence signal intensity of PHEO metastatic organ lesions compared to the PBS-treated group. Finally, the crucial role of CD8^+^ cells in the inhibition of bioluminescence signal intensity of metastatic organ lesions was further supported by the antibody-dependent CD4^+^/CD8^+^ cell depletion experiment.

## 2. Results

### 2.1. A Syngeneic PHEO Mouse Model for Immunotherapy Evaluation

**Figure 2:**
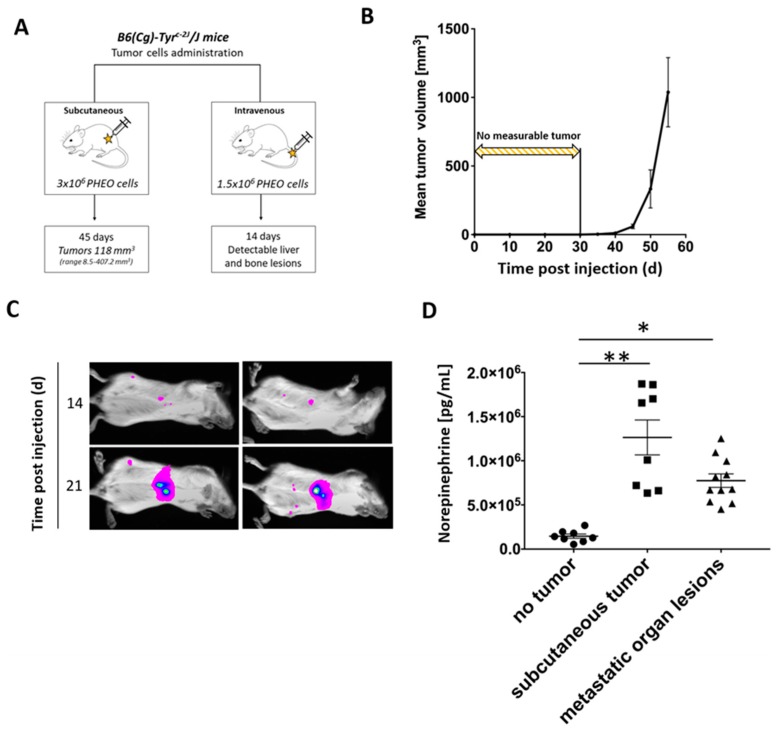
Subcutaneous and metastatic PHEO in mouse model suitable for immunotherapy testing established using MTT-luciferase cells. (**A**) B6(Cg)-Tyr^c-2J^/J mice were subcutaneously (*n* = 24) or intravenously (*n* = 10) injected with MTT-luciferase cells. (**B**) Subcutaneous MTT-luciferase tumors reached a mean volume of 118 mm^3^ 45 days after tumor cell injection. No tumors were detected for 30 days after tumor cells injection. (**C**) Metastatic organ lesions were detectable 14 days after intravenous tumor cells injection using bioluminescence imaging. Metastatic organ lesions were predominantly located in the liver; small lesions were also detected in bones and lymph nodes. (**D**) Tumor-bearing mice, either with subcutaneous tumors or metastatic organ lesions, had significantly higher urine norepinephrine levels than those without tumors (* *p* < 0.05; ** *p* < 0.01 against no tumor).

Since there is a very limited number of animal models for PHEO, we first decided to establish a PHEO mouse model that is appropriate for immunotherapy evaluation. Female B6(Cg)-*Tyr^c-2J^*/J mice were subcutaneously injected with 3 × 10^6^ MTT-luciferase cells in 0.2 mL of DMEM without additives in the right lower dorsal site or intravenously with 1.5 × 10^6^ MTT-luciferase cells in 0.1 mL of PBS in a lateral tail vein. Subcutaneous PHEO tumors reached a mean volume of 118 mm^3^ (range 8.5–407.2 mm^3^) 45 days after tumor cell injection. Mice with intravenously injected tumor cells had detectable metastatic organ lesions 14 days after tumor cell transplantation. These metastatic organ lesions were located predominantly in the liver; small lesions were also detected in bones and lymph nodes (Figure 2A).

Subcutaneous PHEO tumors were not measurable until 30 days after tumor cell injection. However, 40 days after tumor cell injection, the tumor volume increased significantly (Figure 2B). A diameter size of 2 cm was reached after 57 days (range 49–85 days) in most of the experimental mice. Subcutaneous tumors development was observed in 100% of mice. Metastatic organ lesions, after intravenous PHEO tumor cell injection, were detectable in 65% of mice 14 days after tumor cell injection using bioluminescence imaging. On the 21st day, 95% of mice had detectable metastatic liver lesions (Figure 2C).

To further characterize the established PHEO mouse model, urine catecholamine levels (norepinephrine, dopamine, and epinephrine) were measured in B6(Cg)-*Tyr^c-2J^*/J tumor-bearing mice (subcutaneous tumor and metastatic organ lesions) after the injection of MTT-luciferase cells. Norepinephrine levels were significantly higher in tumor-bearing mice (subcutaneous tumor and metastatic organ lesions) compared to non-tumor-bearing mice (no tumor) (Figure 2D). Dopamine levels did not reveal any significant changes when comparing groups of tumor-bearing mice (subcutaneous tumor and metastatic organ lesions) to non-tumor-bearing mice (no tumor) (Figure S1A). Epinephrine levels were higher in mice with metastatic organ lesions compared to non-tumor-bearing mice (no tumor) and tumor-bearing mice (subcutaneous tumor) (Figure S1B).

### 2.2. MBT Immunotherapy Stabilize Tumor Volume and Improves Mice Survival

**Figure 3:**
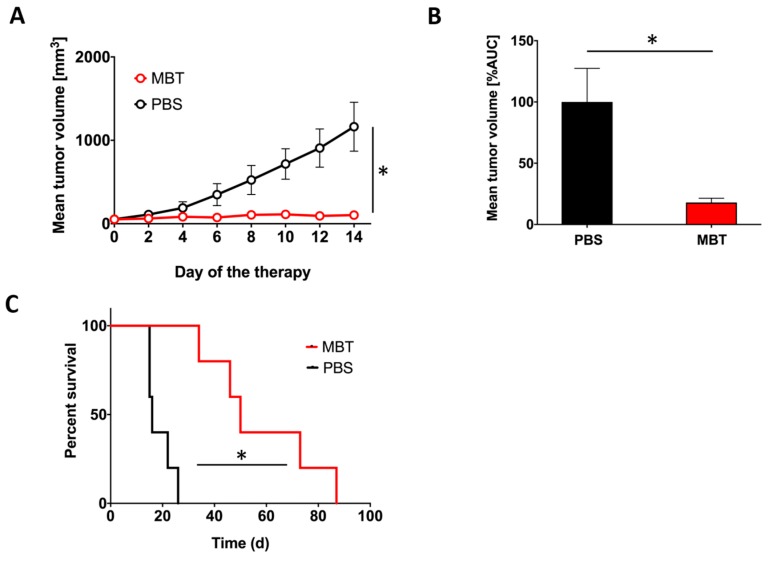
Intratumoral application of MBT in subcutaneous PHEO. B6(Cg)-*Tyr*^c-2J^/J mice were subcutaneously injected with MTT-luciferase cells. After tumors grew to the desired size (about 100 mm^3^), mice were randomized into two groups (*n* = 5/group): (i) the group treated with MBT; (ii) the group treated with PBS. MBT and PBS were given intratumorally on days 0, 1, 2, 8, 9, 10, 16, 17, 18, 24, 25, and 26. Tumor volume was measured with a caliper. (**A**) The tumor volume growth is presented as a growth curve (* *p* < 0.05 against PBS) and (**B**) as an area under the curve (AUC) (* *p* < 0.05 against PBS). (**C**) The survival analysis for the two groups are presented as a Kaplan–Meier curve (* *p* < 0.05 against PBS).

To evaluate the effect of MBT in PHEO, we applied MBT into subcutaneous PHEO tumors (on specific days as described in Materials and Methods). MBT application resulted in a significant stabilization of subcutaneous PHEO volume compared to the PBS-treated group (Figure 3A,B).

In addition, intratumoral application of MBT resulted in significantly longer survival of the treated mice. The median survival increased from 16 days (range 15–26 days) in the PBS-treated group to 50 days (range 34–87 days) in the MBT-treated group (Figure 3C).

### 2.3. Significant Participation of Innate Immunity in Subcutaneous PHEO Volume Stabilization

**Figure 4:**
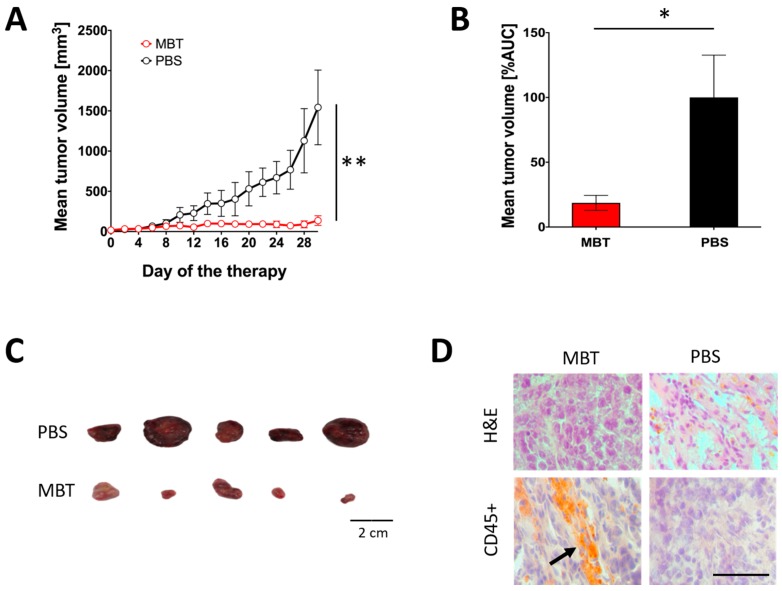
Significance of innate immunity in MBT immunotherapy in subcutaneous PHEO. B6.CB17-Prkdc^scid^/SzJ mice were subcutaneously injected with MTT-luciferase cells. After tumors grew to the desired size (about 50 mm^3^), mice were randomized into two groups (*n* = 6/group): (i) the group treated with MBT and (ii) the group treated with PBS. MBT and PBS were given intratumorally on days 0, 1, 2, 8, 9, 10, 16, 17, 18, 24, 25, and 26. Tumor volume was measured with a caliper. (**A**) The tumor volume growth is presented as a growth curve (** *p* < 0.01 against PBS) and (**B**) as an area under the curve (AUC) (* *p* < 0.05 against PBS). (**C**) Surviving mice were sacrificed on the 30th day of therapy (five mice from the MBT-treated group and five mice from the PBS-treated group), and tumors were documented. (**D**) Hematoxylin and eosin (H&E) staining and CD45^+^ immunohistochemistry staining were performed on tumor cryosections (a thickness of 8 µm). Bar = 20 µm.

In the subsequent experiment, we used B6.CB17-*Prkdc^scid^*/SzJ mice lacking functional T and B cells to verify the role of innate and adaptive immunity in the PHEO volume stabilization during MBT therapy. Subcutaneous PHEOs were treated intratumorally with MBT or PBS on specific days as described in Materials and Methods. Intratumoral application of MBT resulted in PHEO volume stabilization compared to the PBS-treated group (Figure 4A,B). Since we started this experiment with a lower tumor volume than in the previous experiment (about 50 mm^3^), most of the experimental mice (five of six mice from both the MBT- and PBS-treated groups) survived during the whole course of the therapy (30 days). On the 30th day of therapy, mice from both groups were sacrificed, and subcutaneous tumors were harvested and then analyzed. The MBT-treated tumors were significantly smaller than the PBS-treated tumors (Figure 4C). Additional immunohistochemical analysis showed higher levels of tumor-infiltrating leukocytes (CD45^+^ cells) in the MBT-treated group compared to the PBS-treated group (Figure 4D).

### 2.4. Characterization of Tumor-Infiltrating Leukocytes and Tumor Environment during MBT Immunotherapy

#### 2.4.1. Flow Cytometry Analysis of Tumor-Infiltrating Leukocytes in the MBT-Treated Tumors

**Figure 5:**
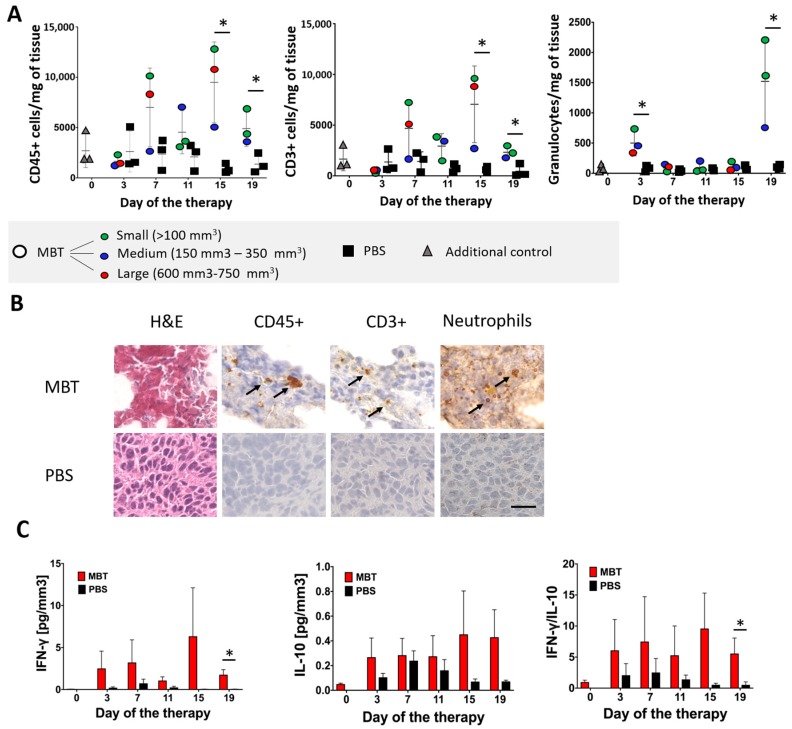
Flow cytometry and immunohistochemistry analysis of tumor-infiltrating leukocytes in the MBT-treated tumors. B6(Cg)-*Tyr*^c-2J^/J mice were subcutaneously injected with MTT-luciferase cells. After tumors grew to the desired size (about 100 mm^3^), mice were randomized into two groups (*n* = 24/group): (i) the group treated with MBT and (ii) the group treated with PBS. MBT and PBS were given intratumorally on days 0, 1, 2, 8, 9, 10, 16, 17, 18, 24, 25, and 26. Three mice from both groups were sacrificed on days 3, 7, 11, 15, and 19. One half of the harvested subcutaneous tumors was used for flow cytometry analysis of tumor-infiltrating leukocytes and the second half was used for immunohistochemistry analysis of tumor-infiltrating leukocytes. Three mice were sacrificed on day 0 and used as an additional control—gray triangles (no application of any compounds into the tumor). (**A**) The analysis of tumor-infiltrating CD45^+^ and CD3 ^+^ cells revealed their elevation on the 15th and 19th days of therapy in the MBT-treated group. Granulocytes (Ly6G^+^ cells) were elevated on the 3rd and 19th days of therapy in the MBT-treated group. The results are presented as individual values for each mouse, with a color legend based on the size of the analyzed tumors. (* *p* < 0.05 against PBS). (**B**) Hematoxylin and eosin (H&E) staining and CD45^+^, CD3^+^, and Ly6G/Ly6C immunohistochemistry staining were performed on tumor cryosections (a thickness of 8 µm). Bar = 20 µm. (**C**) IFN-γ levels, measured by ELISA from tumor supernatants collected during tumor-infiltrating leukocyte analysis revealed significantly higher levels in the MBT-treated group compared to the PBS-treated group. The IL-10 analysis revealed low levels in both the MBT-treated group and the PBS-treated group. The IFN-γ/IL-10 ratio was significantly higher in the MBT-treated group compared to the PBS-treated group (* *p* < 0.05 against PBS).

To identify immune cells infiltrating subcutaneous PHEO tumors during MBT therapy, we performed a flow cytometry analysis of tumor-infiltrating leukocytes. Since we limited the number of mice per group (*n* = 3/group), we decided to present tumor-infiltrating leukocytes data as individual values for each mouse, with a color legend based on the size of the analyzed tumors. The flow cytometry analysis of tumor-infiltrating leukocytes showed increased levels of CD45^+^ cells in the MBT-treated group. This trend culminated on the 15th day of therapy (Figure 5A). T cells (CD3^+^) were the most common leukocytes in the MBT-treated tumors (Figure 5A). The analysis of CD3^+^ subpopulations revealed increased levels of Th cells (CD4^+^) (Figure S2A) and Tc cells (CD8^+^) (Figure S2B) in the MBT-treated group. Furthermore, a significant increase in granulocytes was observed on the 3rd and 19th days of therapy (Figure 5A). No significant changes were observed in B cells (CD19^+^) (Figure S2C), monocytes/macrophages (F4/80^+^) (Figure S2D), or natural killer (NK) cells (Figure S2E).

#### 2.4.2. Histological Analysis of Tumor-Infiltrating Leukocytes in the MBT-Treated Tumors

To verify the flow cytometry results of tumor-infiltrating leukocytes, we performed hematoxylin and eosin (H&E) staining and immunohistochemistry staining on the same tumors, which were originally used for the flow cytometry analysis (Figure 5B). Tumors harvested on the 19th day of therapy are presented in Figure 5B as a representative example of infiltrating CD45^+^ cells and their subpopulations. H&E staining showed extensive necrotic areas in tumor tissues in the MBT-treated group. In contrast, no or very small necrotic areas were detected in the PBS-treated group. CD45^+^ immunostaining revealed higher levels of tumor-infiltrating leukocytes during the whole course of therapy in the MBT-treated group compared to the PBS-treated group (Figure 5B). CD45^+^ cells were predominantly localized in necrotic areas of the tumor. Furthermore, CD3^+^ immunohistochemistry staining revealed higher T cell infiltration in the MBT-treated group during the entire course of therapy compared to the PBS-treated group (Figure 5B). Ly6G/Ly6C immunostaining revealed increased infiltration of neutrophils on the 19th day of therapy in the MBT-treated group (Figure 5B).

#### 2.4.3. Interferon Gamma (IFN-γ) and Interleukin 10 (IL-10) Levels Detection in the MBT-Treated Tumors

The high levels of IFN-γ (Figure 5C), low levels of IL-10 (Figure 5C), and high ratio of IFN-γ/IL-10 (Figure 5C) revealed a Th1 shift in the tumor microenvironment in the MBT-treated tumors.

### 2.5. In Vitro Analysis of Neutrophil Cytotoxic Effects toward PHEO Cells and Neutrophil-PHEO Cell Interactions Based on Labeling of Tumor Cells with Mannan-BAM

**Figure 6:**
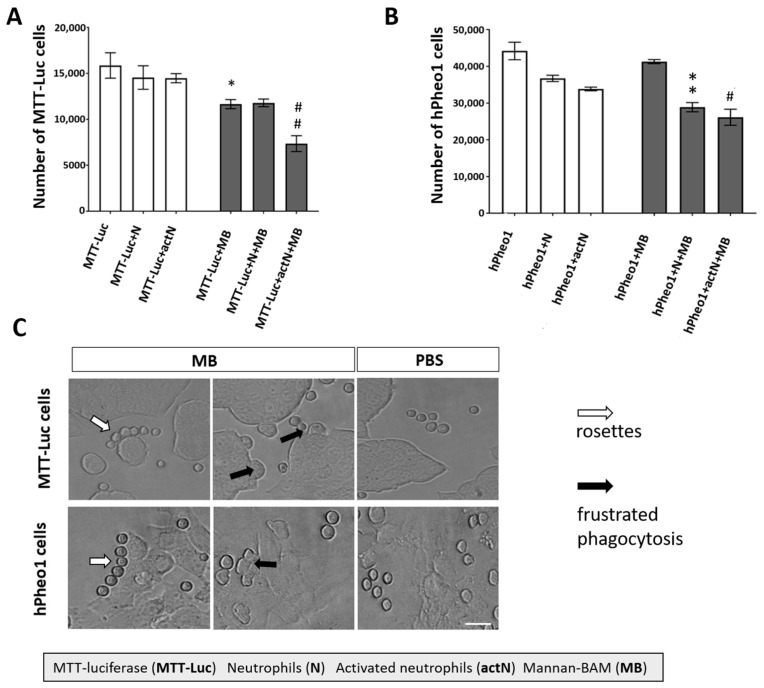
Neutrophil cytotoxicity against PHEO cells labeled with mannan-BAM and neutrophil-PHEO cell interactions. Mouse neutrophils (isolated from bone marrow of B6(Cg)-*Tyr*^c-2J^/J non-tumor-bearing mice) and human neutrophils (isolated from whole blood of healthy donors) were activated by cytokines (granulocyte-macrophages colony-stimulatory factor (GM-CSF), tumor necrosis factor alpha (TNFα), and laminarin) and cultivated with MTT-luciferase and hPheo1 tumor cells with or without mannan-BAM attached to their surface. Mouse neutrophils were mixed with MTT-luciferase cells and human neutrophils were mixed with hPheo1 cells. After two hours of incubation, neutrophils were stained with anti-mouse or anti-human CD45 antibody. One microliter of DAPI was used for the staining of dead cells. Live tumor cells were measured using a BD FACSCanto II analyzer and evaluated using FlowJo software. (**A**) Analysis of mouse neutrophil cytotoxicity against MTT-luciferase cells revealed a statistically significant increase in neutrophil cytotoxicity toward MTT-luciferase cells with mannan-BAM attached to the tumor cell membrane (* *p* < 0.05 against MTT-luciferase (MTT-Luc) group, ## *p <* 0.01 against MTT-luciferase activated neutrophils group (MTT-Luc+actN)). (**B**) Analysis of human neutrophil cytotoxicity against hPheo1 cells revealed a statistically significant increase in neutrophil cytotoxicity toward hPheo1 cells with mannan-BAM attached to the tumor cell membrane (** *p* < 0.01 against hPheo1+neutrophils group (hPheo1+N), # *p* < 0.05 against hPheo1+activated neutrophils group (hPheo+actN)). (**C**) Frustrated phagocytosis (black arrows) and neutrophil rosettes (white arrows) were detected in the group with mannan-BAM attached to the tumor cell membrane. Bar = 100 µm.

To verify the positive effect of mannan-BAM binding to PHEO cells on their recognition by innate immune cells, we measured (i) cytotoxic activity of neutrophils on PHEO cells with or without mannan-BAM and (ii) neutrophil-PHEO cell interactions. The cytotoxic experiments using PHEO cell lines (MTT-luciferase and hPheo1) revealed an increased cytotoxic effect of neutrophils toward PHEO cells labeled by mannan-BAM compared to the cells without mannan-BAM (Figure 6A,B). Microscopic evaluation of neutrophils and mannan-BAM-labeled PHEO cells showed enhanced frustrated phagocytosis and neutrophil rosette formation in the mannan-BAM group (Figure 6C).

### 2.6. Anti-CD40 Addition Improved Survival in the MBT-Treated Mice

**Figure 7:**
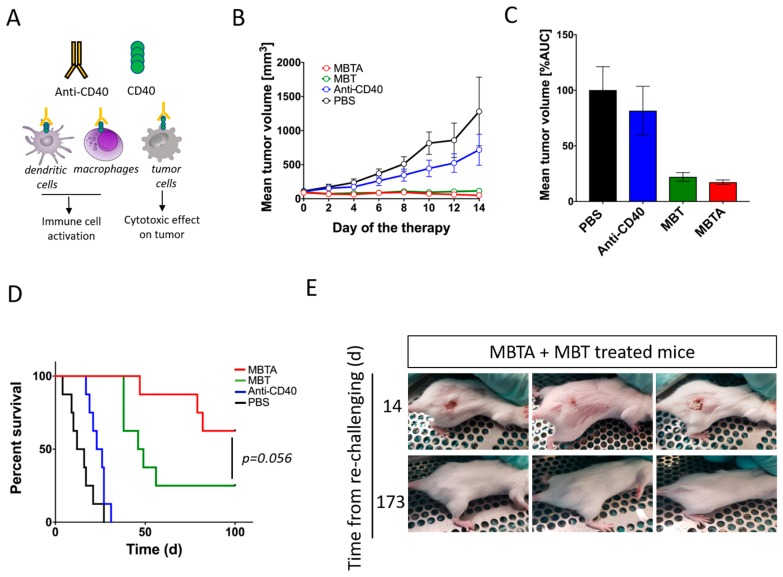
The effect of anti-CD40 addition into MBT therapeutic mixture and re-challenge experiment. B6(Cg)-*Tyr*^c-2J^/J mice were subcutaneously injected with MTT-luciferase cells. After tumors grew to the desired size (about 100 mm^3^), mice were randomized into four groups (*n* = 8/group): (i) the group treated with MBTA, (ii) the group treated with MBT, (iii) the group treated with anti-CD40, and (iv) the group treated with PBS. Therapy was given intratumorally on days 0, 1, 2, 8, 9, 10, 16, 17, 18, 24, 25, and 26. (**A**) Anti-CD40 is an agonist antibody binding to transmembrane protein CD40. CD40 is expressed on variety of cells, such as macrophages, dendritic cells, and some tumor cells. (**B**) The tumor volume growth is presented as a growth curve and (**C**) as an area under the curve (AUC). (**D**) The survival analysis is presented as a Kaplan–Meier curve (*p* = 0.056). (**E**) Mice with complete tumor elimination from the groups treated with MBTA (*n* = 5) and with MBT (*n* = 2) were re-challenged on day 120 (since the start of the therapy) by 3 × 10^6^ MTT-luciferase cells. All animals rejected injected tumor cells.

In order to increase the therapeutic effect of MBT in the PHEO mouse model, we decided to combine MBT with an immunostimulatory monoclonal antibody: anti-CD40. Anti-CD40 is an agonist antibody binding to CD40 transmembrane protein expressed on a variety of cells such as macrophages, dendritic cells, and some tumor cells. The interaction of anti-CD40 with CD40 on the surface of immune cells supports their activation and enhances the immune response (Figure 7A). Interestingly, the beneficial effect of anti-CD40 addition into the MBT therapeutic mixture was not evident during the first 14 days of the therapy (Figure 7B,C). However, the combination of MBT with anti-CD40 (MBTA), in the long term, increased mice survival when compared to the group treated only with MBT (Figure 7D). Moreover, five of eight mice from the MBTA-treated group manifested a complete elimination of subcutaneous tumor, compared to only two of eight mice from the MBT-treated group. A re-challenge experiment, performed with these mice manifested a complete elimination of tumors, revealed resistance against PHEO tumor cell re-injection in both groups, MBT and MBTA (Figure 7E). Interestingly, re-challenged mice initially developed small detectable tumors in the first 14 days; however, after that, all tumors were eradicated with the simultaneous development of skin lesions. These skin lesions were subsequently also eliminated and the whole re-challenged area healed completely.

### 2.7. MBTA Therapy in Metastatic PHEO

**Figure 8:**
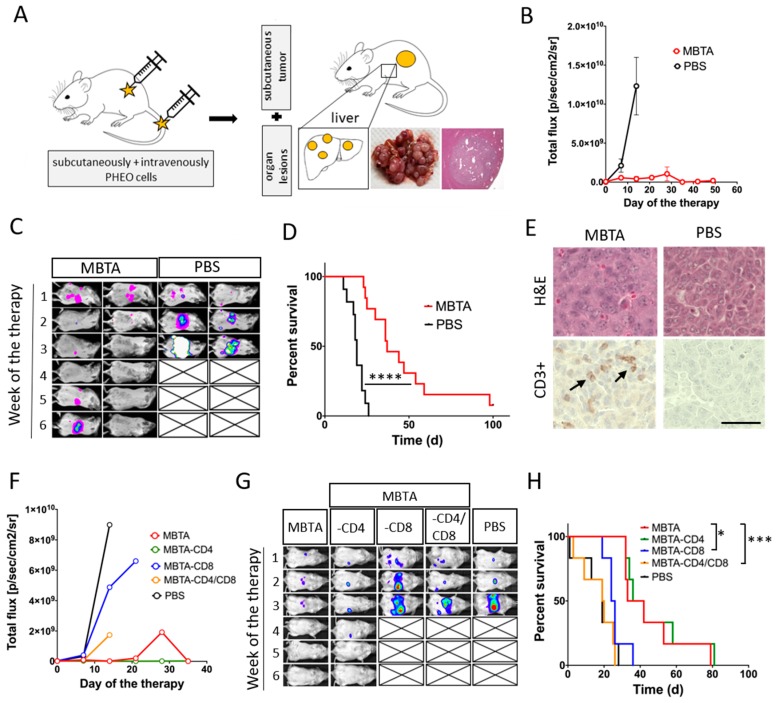
MBTA therapy in metastatic PHEO and the crucial role of CD8^+^ T cells. (**A**) B6(Cg)-*Tyr*^c-2J^/J mice were subcutaneously injected with MTT cells, with subsequent injection of MTT-luciferase cells into the lateral tail vein (2 weeks after subcutaneous injection). When mice developed subcutaneous tumors (around 250 mm^3^) together with metastatic lesions (predominantly in the liver), they were randomized into two groups: (i) the group treated with MBTA (*n* = 13) and (ii) the group treated with PBS (*n* = 12). Therapy was given intratumorally on days 0, 1, 2, 8, 9, 10, 16, 17, 18, 24, 25, and 26. (**B**,**C**) An in vivo bioluminescence assay showed bioluminescence signal intensity inhibition of metastatic organ lesions in the MBTA-treated group compared to the PBS-treated group (p/sec/cm^2^/sr = photons/second/cm^2^/steradian). (**D**) The survival analysis is presented as a Kaplan–Meier curve (**** *p* < 0.0001 against PBS). (**E**) Immunohistochemistry analysis of CD3^+^ cells in metastatic organ lesions in the MBTA- and the PBS-treated group revealed strong infiltration by CD3^+^ cells in the MBTA-treated group. Bar = 20 µm. For the CD4^+^ and CD8^+^ T cell depletion experiment, mice with both subcutaneous PHEO tumor and metastatic organ lesions were randomized equally into five groups (*n* = 6/group): (a) the group treated with MBTA, (b) the group treated with MBTA with intraperitoneal application of anti-CD4, (c) the group treated with MBTA with intraperitoneal application of anti-CD8, (d) the group treated with MBTA with intraperitoneal application of anti-CD4 and anti-CD8, and (e) the group treated with PBS. (**F**,**G**) An in vivo bioluminescence assay showed the important role of CD8^+^ cells in bioluminescence signal intensity inhibition of metastatic organ lesions during MBTA therapy. Part F is presented without SEM as a result of extensive overlap of SEM error bars. (**H**) The survival analysis is presented as a Kaplan–Meier curve (* *p* < 0.05, *** *p* < 0.001 against MBTA).

Our results from the re-challenge experiment suggested that MBTA therapy activate not only innate immunity but also adaptive immunity. Therefore, we decided to evaluate MBTA therapy in metastatic PHEO. Metastatic PHEO was established by prior subcutaneous injection of MTT cells into the right flank of experimental mice followed by intravenous injection of MTT-luciferase cells into the lateral tail vein (2 weeks after subcutaneous injection). These mice, which developed both subcutaneous tumors as well as metastatic organ lesions (predominantly in the liver), were selected for the subsequent experiment (Figure 8A).

When MBTA therapy was tested in this metastatic PHEO (MBTA was applied intratumorally into subcutaneous tumors), we detected lower bioluminescence signal intensity of metastatic organ lesions in the MBTA-treated group compared to the PBS-treated group (Figure 8B,C). In addition, the survival in the MBTA-treated group increased significantly (median survival: 37 days) compared to the PBS-treated group (median survival: 19 days, *p* < 0.0001). Moreover, one mouse from the MBTA-treated group survived for more than 100 days since the beginning of the therapy and manifested a complete regression of metastatic organ lesions (Figure 8D). Histologic sections of metastatic liver lesions showed stronger T cell (CD3^+^) infiltration in the MBTA-treated group compared to the PBS-treated group (Figure 8E).

We further evaluated the role of T cells, specifically CD4^+^ and CD8^+^ T cells, in MBTA therapy. The CD4^+^ and CD8^+^ T cell depletion in metastatic PHEO revealed the importance of CD8^+^ T cells in inhibition of bioluminescence signal intensity of metastatic organ lesions (Figure 8F,G). When CD8^+^ T cells, alone or simultaneously with CD4+ T cells, were depleted in the MBTA-treated group, the bioluminescence signal intensity of metastatic organ lesions was comparable to the PBS-treated group (Figure 8F,G). The same effect was reflected in survival analysis where the depletion of CD8^+^ T cells alone (MBTA-CD8) or with CD4^+^ T cells (MBTA-CD4/CD8) significantly decreased the survival of treated mice (Figure 8H).

## 3. Discussion

In the present study, we showed that the application of mannan anchored to a tumor cell membrane via BAM along with TLR ligands (R-848, poly(I:C), LTA) (a combination referred as MBT) resulted in the stabilization of subcutaneous PHEO volume and significantly improved mice survival. The crucial role of initial activation of innate immunity during MBT therapy was further verified using B6.CB17-*Prkdc^scid^*/SzJ mice lacking functional T and B cells. Similar to B6(Cg)-*Tyr*^c-2J^/J mice, in B6.CB17-*Prkdc^scid^*/SzJ mice, the subcutaneous PHEO volume remained stable in the MBT-treated group compared to the PBS-treated group. Flow cytometry analysis of tumor-infiltrating leukocytes and in vitro experiments in this model showed the potential role of granulocytes (specifically neutrophils) in innate immunity-induced PHEO elimination. An additional combination of MBT with agonistic anti-CD40 antibody (MBTA) resulted in increased mice survival and increased incidence of complete subcutaneous PHEO elimination. Interestingly, a re-challenge experiment in animals with the complete elimination of subcutaneous PHEO showed a generation of an excellent memory immune response with subsequent rejection of MTT-luciferase cells. To verify the activation of specific immunity (which was suggested by the observed immune memory response), we performed an experiment in a metastatic PHEO mouse model, where MBTA therapy resulted in lower bioluminescence signal intensity of metastatic organ lesions compared to the PBS-treated group. The subsequent CD4^+^ and CD8^+^ T cell depletion experiment confirmed the role of CD8^+^ T cells in this bioluminescence signal intensity inhibition of metastatic organ lesions.

As a first step, we developed a mouse model of PHEO for immunotherapy testing with an option to inject PHEO tumor cells subcutaneously or intravenously. We used the B6(Cg)-*Tyr^c-2J^*/J mouse strain injected with MTT or MTT-luciferase cells. MTT cells were originally developed from liver metastases arising from MPC cells injected intravenously [31]. Moreover, this PHEO mouse model is known to release catecholamines from experimental tumors resembling PHEOs found in patients. However, there are two main limitations arising from the practical use of this PHEO mouse model: (i) a long waiting period from tumor cell injection to tumor formation and (ii) very inconsistent tumor growth, which caused difficulties with the randomization of mice into the groups, resulting in lower numbers of mice per group.

After establishing a subcutaneous PHEO mouse model, we initiated the evaluation of MBT immunotherapy in PHEO. The MBT immunotherapy was previously tested in a melanoma mouse model and a very challenging pancreatic adenocarcinoma mouse model. Specifically, in the melanoma mouse model, MBT immunotherapy resulted in an 83% survival rate of treated mice with a potential anti-metastatic effect [17]. In pancreatic adenocarcinoma, MBT immunotherapy resulted in the suppression of metastases growth, but no increase in the survival rate of treated mice was detected [17]. In a PHEO mouse model, MBT therapy resulted in a subcutaneous PHEO volume stabilization compared to the PBS-treated group. The strategy of promoting an anti-tumor immune response using TLR ligands is well known and was previously successfully tested in many types of tumors [32,33,34]. However, our concept is unique, because of the specific combination of TLR ligands (particularly TLR2, TLR3, and TLR7/8), which seems to have an extraordinary effect on innate immunity activation and tumor elimination, as previously presented in melanoma and pancreatic adenocarcinoma mouse models [15,16,17]. Moreover, an additional anti-tumor effect of TLR ligands in this novel concept is provided by a combination with phagocytosis-stimulating ligands, such as mannan, bound to the tumor cell surface [15,16,17].

To further investigate the role of innate and adaptive immunity in the stabilization of subcutaneous PHEO volume during MBT immunotherapy, we used mice lacking functional T and B cells (B6.CB17-Prkdc^scid^/SzJ mice) and, therefore, lacking basic adaptive immunity function. The stabilization of PHEO volume in mice lacking adaptive immunity treated with MBT was comparable to the stabilization of PHEO volume in a mouse model with a fully functional immune system. These results clearly suggest that innate immunity is crucial for stabilization of subcutaneous PHEO volume during MBT immunotherapy.

In order to characterize the underlying innate immunity mechanisms and tumor environment during MBT therapy in subcutaneous PHEO, we analyzed tumor-infiltrating leukocytes in the MBT- and the PBS-treated groups. In the MBT-treated group, we observed a higher level of tumor-infiltrating leukocytes compared to the PBS-treated group. Tumor-infiltrating leukocytes were mainly represented by T cells and granulocytes. As demonstrated in previous experiments with mice lacking functional T and B cells, T cells do not seem to have an important role in the initial elimination of subcutaneous PHEO, so we decided to further focus on the role of granulocytes in this model.

The high ratio of IFN-γ and IL-10 in the MBT-treated tumors indicates that the Th1 polarization of the tumor environment. In general, the tumor environment can be characterized by Th1 or Th2 polarization. Th2 polarization is considered to favor tumor growth (e.g., promoting angiogenesis, inhibiting cell-mediated immunity and tumor cell killing), whereas Th1 polarization exerts antitumor effects [35]. It was also described previously that PHEO/PGL tumors present high levels of M2 macrophage fractions, leading to Th2 polarization and the promotion of tumor angiogenesis [6]. TLRs are known to play crucial roles in immune response polarization. The activation of TLR3 and TLR7 triggers Th1 polarization in a tumor through increased IL-12, IL-23, and type I IFN production [36].

In vitro cytotoxicity experiments confirmed the importance of mannan-BAM bound to the PHEO cell membrane. The decision to use neutrophils, as the most abundant granulocytes, for these in vitro experiments was based on our tumor-infiltrating leukocyte analysis results as described previously. The increase in neutrophil cytotoxicity was dependent on the presence of mannan-BAM attached to the PHEO cells. Moreover, the presence of complement proteins in the tumor-neutrophils reaction environment (ensured by non-heat inactivated fetal bovine serum (FBS) addition) was crucial for the recognition of tumor cells with mannan-BAM and subsequent neutrophil cytotoxicity toward them.

Moreover, the participation of frustrated phagocytosis in PHEO cell elimination was fully dependent on the presence of mannan-BAM. The same findings were observed in the melanoma model when mannan-BAM was used [37].

From the aforementioned results, we concluded that MBT immunotherapy is effective for the stabilization of subcutaneous PHEO volume and for improvement in survival in mouse models with a robust initial activation of innate immunity. However, the biggest challenge in PHEO is metastatic disease. Therefore, in the next part of our study, we focused on how to simultaneously boost activation of adaptive immunity in MBT immunotherapy to achieve a systematic anti-tumor response with subsequent metastatic organ lesion elimination.

In the first step, we chose the anti-CD40 antibody to boost activation of adaptive immunity. The Anti-CD40 agonistic antibody supports the activation of antigen presenting cells, such as B and T cells, dendritic cells, and macrophages, and so establishes an effective humoral and cellular immune response [38]. The combination of anti-CD40 with MBT applied in a pancreatic adenocarcinoma mouse model resulted in an 80% survival rate, which represented significant improvement compared to the MBT therapy without anti-CD40 [17]. In a PHEO mouse model, the combination of the anti-CD40 antibody with MBT increased the incidence of complete elimination of subcutaneous tumors and improved the overall survival of the treated mice. This effect can be explained by the support of tumor antigen presentation and the stronger participation of adaptive immunity via anti-CD40 [39]. A similar effect was previously reported, when the combination of TLR ligands with anti-CD40 significantly stimulated CD8^+^ T cell responses and induced the migration of activated dendritic cells with a promotion in their capacity to present antigens [40,41]. The activation of adaptive immunity with the subsequent generation of a memory immune response was further verified by a re-challenge experiment in animals with a complete elimination of subcutaneous PHEO from the MBT- and MBTA-treated groups. All re-challenged mice rejected injected PHEO cells, which suggests that the MBT-treated mice also manifest partial activation of adaptive immunity. However, anti-CD40 beneficially boosted adaptive immunity, since 62.5% of mice in the MBTA-treated group completely eliminated subcutaneous PHEO compared to only 25% of mice in the MBT-treated group.

To further support our hypothesis of the adaptive immunity activation during MBTA immunotherapy, we tested MBTA immunotherapy in metastatic PHEO. MBTA immunotherapy resulted in a lower bioluminescence signal intensity of metastatic organ lesions compared to the PBS-treated group and in a significant prolongation of mice survival. Moreover, in this experiment, we observed an interesting phenomenon. Mice with fast initial elimination of subcutaneous tumors (complete elimination in the first week of MBTA therapy) manifested decreased bioluminescence signal intensity inhibition of metastatic organ lesions compared to those where subcutaneous tumors persisted during the first three weeks of MBTA therapy. This observation can be explained by insufficient activation of adaptive immunity caused by short-lasting tumor antigen stimulation since the main source of tumor antigens (the subcutaneous tumor) was eliminated very shortly after MBTA therapy initiation. This is also consistent with principles of tumor vaccines, where repeated applications of these vaccines are usually needed to develop a strong adaptive immune response [42,43]. Since we partially predicted this situation, we decided in advance to enroll mice with higher subcutaneous tumor volume (around 250 mm^3^) compared to the previous experiments to provide enough tumor mass for tumor antigen release and adaptive immunity stimulation. The crucial role of T cells in the inhibition of bioluminescence signal intensity of metastatic organ lesions was further verified in the CD4^+^ and CD8^+^ T cell depletion experiment. The depletion of CD8^+^ T cells, alone or both CD4^+^ and CD8^+^ T cells resulted in decreased survival and decreased bioluminescence signal intensity inhibition of metastatic organ lesions compared to the group treated by MTBA without depletion. These finding are consistent with several studies, where the importance of CD8^+^ T cells on visceral disease was also highlighted [44,45].

Although the presented data may be important for future metastatic PHEO/PGL treatment, one important concern must be addressed. This therapy requires a direct application of MBT or MBTA into a tumor. Initially, this could be considered a limitation, particularly for metastatic PHEO/PGL, because metastases are exclusively found in deep organs, lymph nodes, or bones. However, current interventional radiology approaches are capable of treating metastases, even in those problematic locations [46]. Moreover, local therapy offers certain advantages over systemic therapies, such as a delivery of higher concentrations of the drug into the tumor, minimal systemic side effects, no required tumor antigen identification, in situ vaccination by tumor authentic antigens, no pretreatment biopsy, no major histocompatibility complex (MHC) restriction, polyclonal T and B cell stimulation, and low cost [4,47].

We also acknowledge that there are unanswered questions regarding the underlying immune mechanisms during the presented therapy. Therefore, our future directions involve a deeper understanding of adaptive immunity participation during MBTA therapy and its maximal boost for better control of metastatic organ lesion growth. Moreover, a possible combination of MBTA therapy with other therapies will be considered. There may be great potential in the combination of MBTA with checkpoint inhibitors, as MBTA can support the initial infiltration of leukocytes into the tumor or metastatic organ lesions. This can be especially beneficial, since PHEO/PGL are tumors with a low lymphocyte fraction [6] and the prediction for checkpoint inhibitor therapy, as a single treatment, is thus not favorable [48,49,50]. Interestingly, certain subsets of PHEO/PGL tumors showed programed cell death 1 (PD-L1) and programed cell death 2 (PD-L2) expression [51]. In these specific PHEO/PGL subsets, the combination of MBTA with checkpoint inhibitor therapy can potentially intensify the therapeutic response. The doors are also open to a possible combination of MBTA therapy with radiotherapy and/or chemotherapy [52].

## 4. Materials and Methods

### 4.1. Mannan-BAM Synthesis

Mannan-BAM synthesis was performed as previously reported [15,16,17]. Mannan was obtained from Sigma-Aldrich, Saint Louis, MO, USA. BAM was obtained from NOF Corporation, White Plains, NY, USA.

### 4.2. Cell Lines

MTT, MTT-luciferase, and human hPheo1 cell lines were used in this study [30,31]. MTT and MTT-luciferase cells are rapidly growing cells derived from liver metastases of MPC cells, and MTT-luciferase cells are transfected by a luciferase plasmid [30,31]. hPheo1 (obtained from University of Texas, Southwestern Medical Center, Dr. Hans Kumar Ghayee, D.O., MTA# 41611) is a progenitor cell line derived from human PHEO [53].

MTT and MTT-luciferase cells were maintained in Dulbecco’s modified eagle media (DMEM) (Sigma-Aldrich) supplemented with 10% heat-inactivated fetal bovine serum (Gemini, West Sacramento, CA, USA) and penicillin/streptomycin (100 U/mL; Gemini). In MTT-luciferase cells, geneticin (750 μg/mL; Thermo Fisher Scientific, Waltham, MA, USA) was used for stable cell line selection. hPheo1 cells were maintained in RPMI (Sigma-Aldrich) supplemented with 10% heat-inactivated fetal bovine serum and penicillin/streptomycin (100 U/mL). Both cell lines were cultured at 37 °C in humidified air with 5% CO_2_.

All cell lines used were routinely tested for mycoplasma using the MycoAlert^TM^ detection kit (Lonza, Walkersville, MD, USA). Cell authentication was performed per ATCC guidelines using morphology, growth curves, and mycoplasma testing. After thawing, cells were cultured for no longer than 3 weeks.

### 4.3. Establishment of a PHEO Mouse Model and Tumor Cell Injection

Female B6(Cg)-*Tyr^c-2J^*/J and B6.CB17-*Prkdc^scid^*/SzJ mice were purchased from the Jackson Laboratory, Bar Harbor, ME, USA. B6(Cg)-*Tyr^c-2J^*/J mice were used for subcutaneous and metastatic PHEO and represent mouse models with fully functional immune systems. B6.CB17-*Prkdc^scid^*/SzJ mice lacking functional T and B cells were used for subcutaneous PHEO and represent mouse models with dysfunctional adaptive immunity. Mice were housed in specific pathogen-free conditions and all experiments were approved by the *Eunice Kennedy Shriver* National Institute of Child Health and Human Development animal protocol (ASP: 15 028).

For subcutaneous PHEO, B6(Cg)-*Tyr^c-2J^*/J and B6.CB17-*Prkdc^scid^*/SzJ mice were subcutaneously injected in the right lower dorsal site with 3 × 10^6^ and 1.5 × 10^6^ MTT-luciferase cells in 0.2 mL of DMEM without additives, respectively. For metastatic PHEO, B6(Cg)-*Tyr^c-2J^*/J mice were also subcutaneously injected in the right lower dorsal site with 3 × 10^6^ MTT cells in 0.2 mL of DMEM without additives and were simultaneously injected intravenously with 1.5 × 10^6^ MTT-luciferase cells in 0.1 mL of PBS. MTT cells without luciferase where used for the establishment of subcutaneous PHEO to prevent the possibility that the signal from MTT-luciferase cells in large subcutaneous tumors will cover the signal from small metastatic organ lesions during bioluminescence imaging.

For a re-challenge experiment, 3 × 10^6^ MTT-luciferase cells in 0.2 mL of DMEM without additives were injected subcutaneously on day 120 since the beginning of therapy in the animals manifested a complete elimination of tumors due to used immunotherapy.

### 4.4. Treatment

Treatment in subcutaneous PHEO was initiated when subcutaneous tumors reached an average volume of 100 mm^3^ in B6(Cg)-*Tyr^c-2J^*/J mice. In B6.CB17-*Prkdc^scid^*/SzJ mice, lacking functional T and B cells, the treatment was initiated with a lower tumor volume (average volume of 50 mm^3^) to prevent the potential early reach of a tumor endpoint size as a result of the rapid growth of subcutaneous PHEO in mice with a specific immunity disfunction. Treatment in metastatic PHEO was initiated when subcutaneous tumors reached an average volume of 250 mm^3^ with a simultaneous presence of detectable signal in organs using bioluminescence imaging. This greater starting tumor volume was purposely chosen to ensure enough tumor mass for tumor antigen gradual release during the whole course of the therapy. Mice, with both subcutaneous PHEO and metastatic PHEO, were treated intratumorally (into the subcutaneous tumor) on days 0, 1, 2, 8, 9, 10, 16, 17, 18, 24, 25, and 26 with 50 μL of the following mixtures: (a) 0.5 mg of R-848 hydrogen chloride (HCl) (Tocris, Minneapolis, MN, USA), 0.5 mg of poly(I:C) (Sigma-Aldrich), 0.5 mg of LTA/mL (Sigma-Aldrich), and 0.2 mM mannan-BAM (MBT), and later on in combination with anti-CD40, clone FGK4.5/FGK45 (BioXCell, West Lebanon, NH, USA) (MBTA), in PBS; and (b) PBS.

### 4.5. Tumor Size Evaluation

Subcutaneous tumor volume was measured every other day with a caliper and calculated as V = (π/6) AB^2^ (A and B = the largest and the smallest dimension of the tumor, respectively) [54]. Survival curves are based on the time of death caused by tumor growth or on the time of sacrifice of mice reaching the maximally allowed tumor size of 2 cm in diameter. 

In metastatic PHEO, mice were imaged by an IVIS system (Bruker, Billerica, MA, USA) once a week to detect a bioluminescence signal intensity of metastatic organ lesions, and the signal was evaluated using Bruker MI SE software.

### 4.6. Urine Catecholamine Determination

Urine specimens from B6(Cg)-*Tyr^c-2J^*/J tumor-bearing mice were collected after subcutaneous or intravenous injection of MTT-luciferase cells. B6(Cg)-*Tyr^c-2J^*/J mice without tumors were used as controls. All specimens were collected at the same time of the day to prevent any variance in catecholamine levels caused by circadian rhythms. Urine catecholamines (norepinephrine, epinephrine, and dopamine) were analyzed by liquid chromatography with electrochemical detection as described previously [55].

### 4.7. Analysis of Tumor Infiltrating Leukocytes and Spleen Leukocytes

B6(Cg)-*Tyr^c-2J^*/J tumor-bearing mice were euthanized by cervical dislocation. Tumors were harvested from the body. Tumors were washed in cold DMEM and cut into small pieces. For tumor cell dissociation, a tumor dissociation kit (Miltenyi Biotech, Auburn, CA, USA) was used. One hour after incubation in 37 °C with constant agitation, samples were centrifuged. Supernatant was collected and used for the detection of cytokines (interferon gamma (IFN-γ) and interleukin 10 (IL-10)) by an enzyme-linked immunosorbent assay (ELISA). The tumor cell pellet was passed through a 70 µm strainer. The red blood cells were removed using ammonium-chloride-potassium (ACK) lysing buffer (Thermo Fisher Scientific). Leukocytes (CD45^+^ cells) and their subpopulations were stained using the following antibodies: APC anti-mouse CD45, clone: 30-F11 (BioLegend, Dedham, MA, USA); Brilliant Violet 650 anti-mouse CD19, clone: 6D5 (BioLegend); FITC anti-mouse CD3, clone: 17A2 (BioLegend); Brilliant Violet 605 anti-mouse CD4, clone: RM4-5 (BioLegend); APC/Cy7 anti-mouse CD8a, clone: 53-6.7 (BioLegend); PE/Cy7 anti-mouse Ly-6G/Ly-6C (GR-1), clone: RB6-8C5 (BioLegend); PE anti-mouse NK-1.1, clone: PK136 (BioLegend); and Brilliant Violet 421 anti-mouse F4/80, clone: BM8 (BioLegend). LIVE/DEAD^®^ fixable yellow dead cell stain (Invitrogen, Carlsbad, CA, USA) was used to eliminate dead cells. Samples were measured using a BD Fortessa analyzer (San Jose, CA, USA) and evaluated with FlowJo software (Ashland, OR, USA). CountBright absolute counting beads (Invitrogen) were used to count absolute numbers of individual CD45^+^ cells.

### 4.8. Cytokine Assay

Supernatant collected during the analysis of tumor infiltrating leukocytes was used to measure INF-γ and IL-10 levels in the tumors during the therapy. The following ELISA kits were used for the detection: IFN-γ Mouse ELISA Kit, Extra Sensitive (Thermo Fisher), and Mouse IL-10 ELISA Kit (LSBio, Seattle, WA, USA).

### 4.9. Immunohistochemistry

Tumor tissue samples embedded in Tissue-Tek optimum cutting temperature (OCT) were sectioned by a microtome-cryostat (8 μm). Formalin fixed, paraffin-embedded tissue specimen was prepared for 5 μm sections. Frozen sections were fixed in HistoChoice MB tissue Fixative solution (Ambresco, Cleveland, OH, USA). Subsequently in both frozen and paraffin-embedded sections, peroxidase activity was inhibited by 3% hydrogen peroxide. Additionally, samples were blocked using SuperBlock blocking buffer (Thermo Fisher Scientific). Anti-mouse CD45 antibody, anti-mouse Ly6G/Ly6C antibody, and anti-mouse CD3 antibody (Abcam, Cambridge, MA, USA) were used for immunohistochemistry staining. The signal was developed by diaminobenzidine (DAB) substrate (Dako, Santa Clara, CA, USA).

### 4.10. Neutrophil Cytotoxicity toward PHEO Cells

Bone marrow from B6(Cg)-*Tyr^c-2J^*/J non-tumor-bearing mice was used as a source of mouse neutrophils. Bone marrow isolation was performed according to Stassen et al. [56]. Untouched mouse neutrophils were isolated by magnetic-activated cell sorting using a neutrophil isolation kit (Miltenyi Biotec). Human neutrophils were isolated from whole blood of healthy donors received from the National Institutes of Health (NIH) Blood Bank using an EasySep Direct Human Neutrophils Isolation kit (Stemcell Technologies, Cambridge, MA, USA). Both human and mouse neutrophils were activated for 20 min by a mixture of GM-CSF (12 ng/mL) (Sigma-Aldrich), TNFα (2.5 ng/mL) (Sigma-Aldrich), and 2 μM laminarin (Sigma) as previously described [57].

MTT-luciferase or hPheo1 cells were incubated with 0.02 mM mannan-BAM in culture medium for 30 min in 37 °C. After incubation and washing by centrifugation, mouse MTT-luciferase cells were co-cultured with activated or non-activated murine neutrophils and human hPheo1 cells with activated or non-inactivated human neutrophils for 2 h in 37 °C. The tumor cell/neutrophil ratio was 1:5 (50,000 tumor cells to 250,000 neutrophils). Dead cells were stained with DAPI (1 μM) (Invitrogen). APC anti-mouse CD45 antibody, clone 30-F11 (BioLegend), and APC anti-human CD45 antibody, clone H130 (BioLegend), were used to stain leukocytes (in this specific case neutrophils). CountBright absolute counting beads (Invitrogen) were used to count absolute numbers of live tumor cells in the samples. Samples were measured by FACSCantoII. FlowJo software was used for analysis.

### 4.11. Imaging of PHEO Cell–Neutrophil Interactions

Adhered hPheo1 or MTT-luciferase cells were incubated with 0.02 mM mannan-BAM in culture medium for 30 min in 37 °C. Following incubation, unbound mannan-BAM was washed by centrifugation and human neutrophils were added to the hPheo1 cells and mouse neutrophils to the MTT-luciferase cells (the ratio of tumor cells to neutrophils was 1:2 (50,000 tumor cells:100,000 neutrophils). Neutrophils and hPheo1/MTT-luciferase cell interactions were documented after 2 h of co-culturing in 37 °C. A Leica DMRB microscope and Leica LAS AF software were used for analysis.

### 4.12. Depletions

Cellular subsets were depleted by administering 300 µg of depleting antibody intraperitoneally twice weekly starting one day prior to immunotherapy: CD8^+^ T-cells with anti-CD8α, clone 2.43 (BioXCell), and CD4^+^ T-cells with anti-CD4, clone GK1.5 (BioXCell). Cellular depletion of CD8^+^ and CD4^+^ T-cells were confirmed by flow cytometry of PBMC blood levels (Figure S3). Samples were measured by FACSCantoII. FlowJo software was used for analysis.

### 4.13. Statistical Analysis

Data were analyzed using STATISTICA 12 (StatSoft, Tulsa, OK, USA) or Prism 7 (GraphPad Software, San Diego, CA, USA). Individual data sets were compared using a dependent/independent Student’s *t*-test. Analyses across multiple groups and times were performed using repeated measures ANOVA (for data with normal distribution) with individual groups assessed using Tukey’s multiple comparison. For data without normal distribution, non-parametric ANOVA was used with individual groups were assessed by Kruskal–Wallis test. Kaplan–Meier survival curves were compared using a log-rank test. Error bars indicate the standard error of the mean (SEM). * *p* < 0.05; ** *p* < 0.01; *** *p* < 0.001; **** *p* < 0.0001. # *p* < 0.05; ## *p* < 0.01.

## 5. Conclusions

We demonstrate here promising therapeutic effects of enhanced innate immunity with subsequent activation of adaptive immunity using intratumoral application of ligands stimulating phagocytosis combined with TLR ligands in subcutaneous and metastatic PHEO in mouse models. This effect was verified in vitro in mouse PHEO and human PHEO cell lines. We suggest that this immunotherapeutic approach could potentially become a novel treatment option in patients with metastatic PHEO/PGL.

## Supplementary Materials

The following are available online at https://www.mdpi.com/2072-6694/11/5/654/s1. Figure S1: Urine dopamine and epinephrine levels in the subcutaneous and metastatic PHEO, Figure S2: Tumor CD4+, CD8+, CD19+, F4/80+, and NK cells levels in the course of MBT therapy, Figure S3: Depletion of CD4+ and CD8+ T cells during MBTA therapy.
