# Supplementary material for: The Significant Reduction or Complete Eradication of Subcutaneous and Metastatic Lesions in a Pheochromocytoma Mouse Model after Immunotherapy Using Mannan-BAM, TLR Ligands, and Anti-CD40
Source: Cancers (Basel). 2019 May 11;11(5):654. doi: 10.3390/cancers11050654 (PMC6562455; doi:10.3390/cancers11050654)
Supplement: Supplementary file 1 [file cancers-11-00654-s001.pdf]

Supplementary Materials

The Significant Reduction or Complete Eradication of Subcutaneous and Metastatic Lesions in a Pheochromocytoma Mouse Model after Immunotherapy Using Mannan-BAM, TLR Ligands, and Anti-CD40

Veronika Caisova, Liping Li, Garima Gupta, Ivana Jochmanova, Abhishek Jha, Ondrej Uher, Thanh-Truc Huynh, Markku Miettinen, Ying Pang, Luma Abunimer, Gang Niu, Xiaoyuan Chen, Hans Kumar Ghayee, David Taïeb, Zhengping Zhuang, Jan Zenka and Karel Pacak

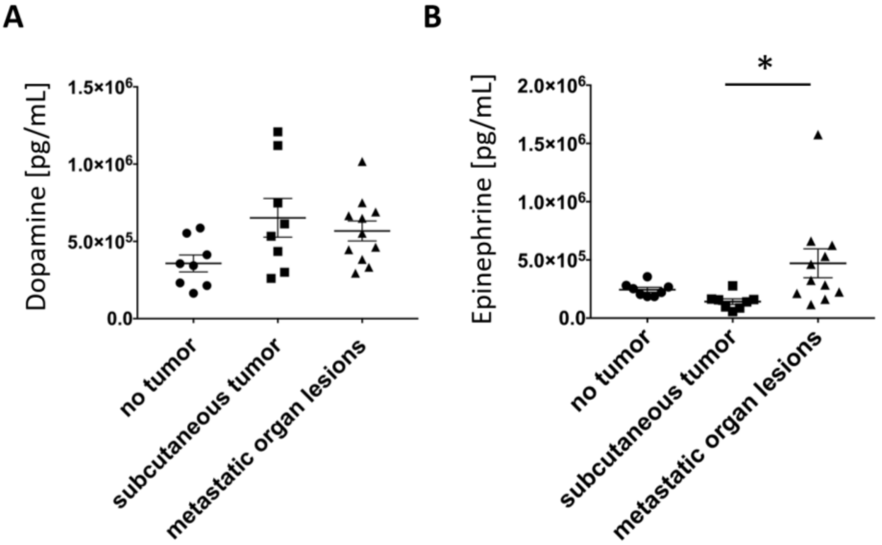

Figure S1. Urine dopamine and epinephrine levels in the subcutaneous and metastatic PHEO. B6(Cg)-*Tyr*c-2J/J ($n = 8$) mice were subcutaneously injected with MTT-luciferase cells in the right lower dorsal site. B6(Cg)-*Tyrc-2J*/J mice ($n = 8$) without tumors were used as controls. (**A**) Urine dopamine levels in tumor-bearing mice were not significantly increased compared to those of non-tumor-bearing mice. (**B**) Urine epinephrine levels in mice with metastatic organ lesions were significantly higher compared to those mice with subcutaneous tumor (* $p < 0.05$ against subcutaneous tumor).

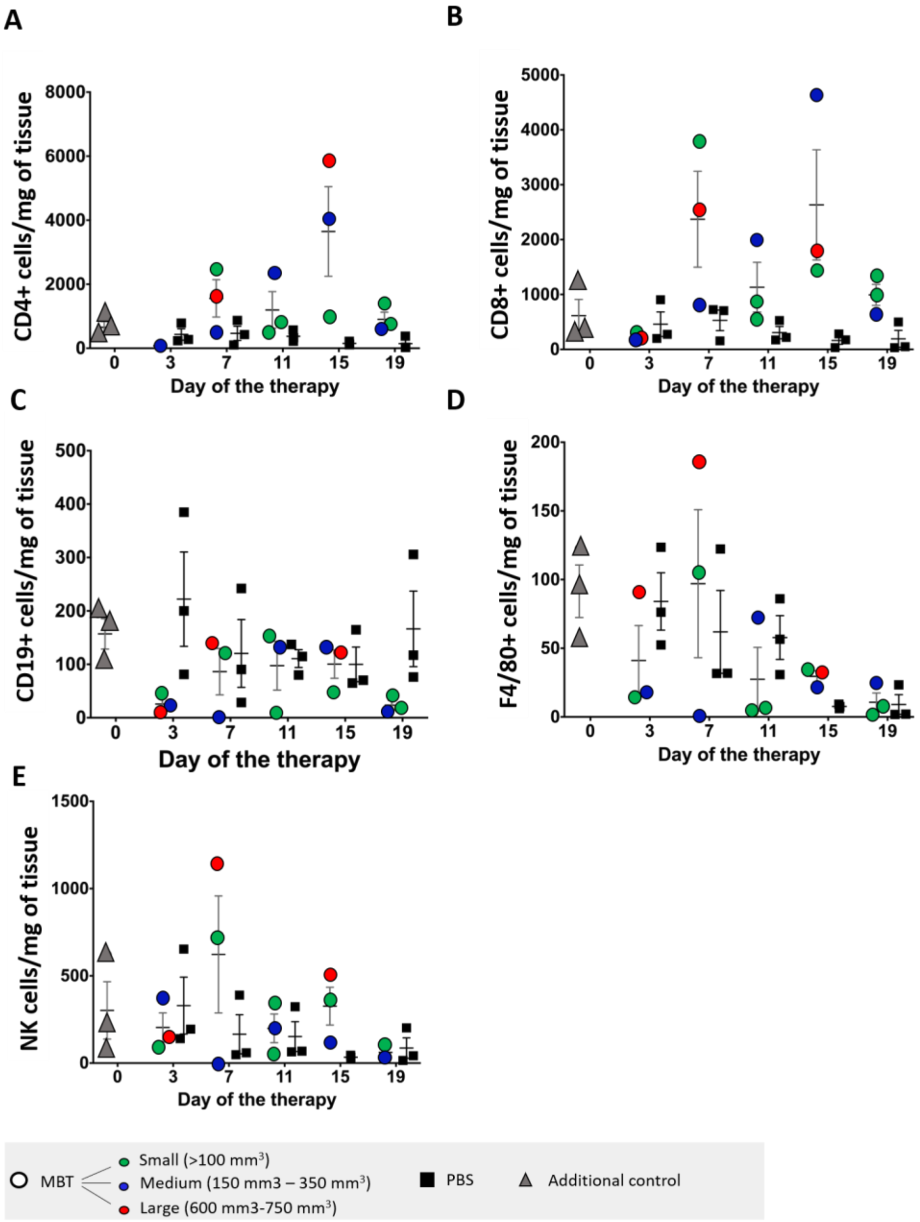

Figure S2. Tumor $CD4^{+}$, $CD8^{+}$, $CD19^{+}$, $F4/80^{+}$, and NK cells levels in the course of MBT therapy. B6(Cg)-*Tyr*$^{c-2J}$/J mice were subcutaneously injected with MTT-luciferase cells. After tumors grew to the desired size (around 100 mm^3), mice were randomized into two groups (*n* = 24/group): (i) the group treated with MBT, (ii) the group treated with PBS. Therapy was given intratumorally on days 0, 1, 2, 8, 9, 10, 16, 17, 18, 24, 25, and 26. Three mice from both groups were sacrificed on days 3, 7, 11, 15, and 19 of the therapy. Harvested subcutaneous tumors were used for flow cytometry analysis of tumor-infiltrating leukocytes. Three mice were sacrificed on day 0 and used as an additional control—gray triangles (no application of any compounds into the tumor). (**A**) The analysis of tumor-infiltrating $CD4^{+}$ cells revealed increased levels of these cells in the MBT-treated group (culminated on the 15th day of therapy). (**B**) The analysis of tumor-infiltrating $CD8^{+}$ cells also revealed increased levels of these cells in the MBT-treated group (culminated on the 15th day of therapy). (**C**) The analysis of tumor-infiltrating $CD19^{+}$ cells did not reveal any significant changes in the MBT-treated group compared to the PBS-treated group. (**D**) The analysis of tumor-infiltrating $F4/80^{+}$ cells did not reveal any significant changes in the MBT-treated group compared to the PBS-treated group. (**E**) The analysis of tumor-infiltrating NK cells did not reveal any significant changes in the MBT-treated group compared to the PBS-treated group. The results are presented as individual values for each mouse with color legend based on the size of analyzed tumors.

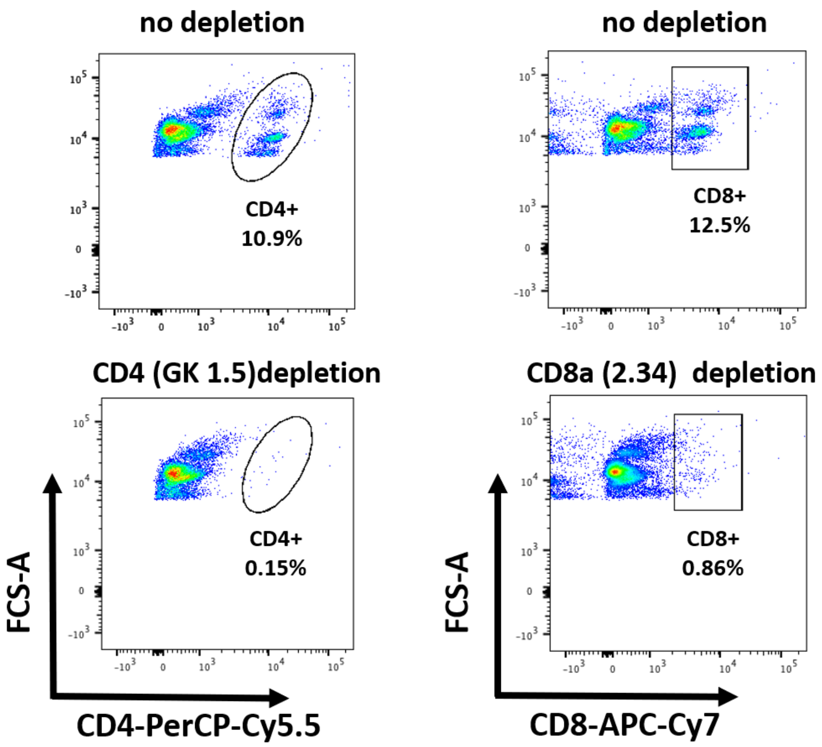

Figure S3. Depletion of $CD4^+$ and $CD8^+$ T cells during MBTA therapy. B6(Cg)-*Tyr*$^{c-2J}$/J mice (*n* = 6/group for $CD4^+$ cells depletion, *n* = 6/group for $CD8^+$ depletion) were subcutaneously injected with MTT-luciferase cells. $CD4^+$ and $CD8^+$ subsets were depleted by administering 300 µg of depleting antibody intraperitoneally twice weekly beginning one day prior to initiation of MBTA therapy. The depletion of $CD8^+$ and $CD4^+$ T cells from PBMCs on day 12 was confirmed using flow cytometry.

© 2019 by the authors. Licensee MDPI, Basel, Switzerland. This article is an open access article distributed under the terms and conditions of the Creative Commons Attribution (CC BY) license (http://creativecommons.org/licenses/by/4.0/).
